# Supplementary material for: Molecular mechanisms of human P2X3 receptor channel activation and modulation by divalent cation bound ATP
Source: eLife. 2019 Jun 24;8:e47060. doi: 10.7554/eLife.47060 (PMC6590987; doi:10.7554/eLife.47060)
Supplement: Supplementary file 1. [file elife-47060-supp1.docx]

Table 1. Data collection, phasing and refinement statistics

|  | Mg^2+-^bound | Ca^2+^-bound |
| --- | --- | --- |
| PDB ID | 6AH5 | 6AH4 |
| **Data collection** |  |  |
| Wavelength (Å) | 1.000 | 1.000 |
| Space group | *C*222_1_ | *C*222_1_ |
| Cell dimensions |  |  |
| *a*, *b*, *c* (Å) | 110.5, 139.4, 324.0 | 110.9, 142.5, 325.3 |
| *α*, *β*, *γ* (°) | 90, 90, 90 | 90, 90, 90 |
| Resolution (Å)^*^ | 50.0 – 3.82 (4.05– 3.82) | 50.0 – 3.30(3.50– 3.30) |
| *R*_sym_^*^ | 0.076 (0.418) | 0.089 (1.100) |
| *I*/σ*I*^*^ | 7.89 (1.94) | 12.77 (1.74) |
| Completeness (%)^*^ | 99.8 (96.1) | 99.9 (99.4) |
| Redundancy^*^ | 3.5 (3.4) | 7.2 (7.4) |
| CC_1/2_ (%)^*^ | 99.8 (96.1) | 99.9 (91.1) |
|  |  |  |
| **Refinement** |  |  |
| Resolution (Å) | 3.82 | 3.3 |
| No. reflections | 46538 | 74823 |
| *R*_work/_ *R*_free_ | 25.6/30.7 | 24.0 /29.0 |
| No. atoms |  |  |
| Protein | 8503 | 8572 |
| ATP | 93 | 93 |
| Cations | 3 | 3 |
| B-factors |  |  |
| Protein | 132.4 | 162.5 |
| ATP | 189.0 | 133.9 |
| Cations | 180.0 | 156.5 |
| R.m.s deviations |  |  |
| Bond lengths (Å) | 0.010 | 0.010 |
| Bond angles (°) | 1.313 | 1.416 |
| Ramachandran plot |  |  |
| Favoured (%) | 94.46 | 94.37 |
| Allowed (%) | 5.54 | 5.63 |
| Outliers (%) | 0 | 0 |
